# 85. Efficacy of Sulbactam-Durlobactam (SUL-DUR) Compared to Colistin (COL) against *Acinetobacter baumannii-calcoaceticus* Complex (ABC) Monomicrobial and Polymicrobial Infections in a Phase 3 Trial

**DOI:** 10.1093/ofid/ofad500.001

**Published:** 2023-11-27

**Authors:** Alita Miller, Sarah McLeod, Adam B Shapiro, Khurram Rana, David Altarac

**Affiliations:** Entasis Therapeutics, Waltham, Massachusetts; Innoviva Specialty Therapeutics, Waltham, Massachusetts; Entasis Therapeutics, Waltham, Massachusetts; Entasis Therapeutics, Waltham, Massachusetts; Entasis Therapeutics, Waltham, Massachusetts

## Abstract

**Background:**

Recently, a global, active-controlled Phase 3 trial evaluated the efficacy and safety of SUL-DUR vs. COL for patients with *Acinetobacter baumannii-calcoaceticus* complex (ABC) infections, including multidrug resistant strains. Both arms were dosed on a background of imipenem/cilastatin (IMI) to treat co-infecting Gram-negative pathogens. 33% of infections in the primary efficacy population were polymicrobial.

**Methods:**

The minimal inhibitory concentrations (MICs) of SUL-DUR, colistin and imipenem against baseline isolates were determined by broth microdilution using CLSI guidelines. The primary efficacy endpoint was 28-day all-cause mortality (ACM) in patients with carbapenem-resistant ABC at baseline (CRABC m-MITT). Clinical and microbiological outcomes were evaluated at Test of Cure (TOC).

**Results:**

28D ACM, clinical and microbiological outcomes were similar for patients in the SUL-DUR arm with either monomicrobial or polymicrobial ABC infections while patients in the COL arm with monomicrobial ABC infections had higher mortality rates with worse clinical and microbiological outcomes compared to those with polymicrobial infections (Table 1). Of the 12 patients in the SUL-DUR arm who did not survive to 28 days, 4 (33.3%) were attributed to the index infection (2 of 6 [33.3%] for both monomicrobial and polymicrobial ABC infections). Of the 20 patients in the COL arm who did not survive to 28 days, 10 (50%) were attributed to the index infection (7 of 15 [46.7%] with monomicrobial ABC infections and 3 of 5 [60%] with polymicrobial ABC infections) (Table 2).

**Figure:**
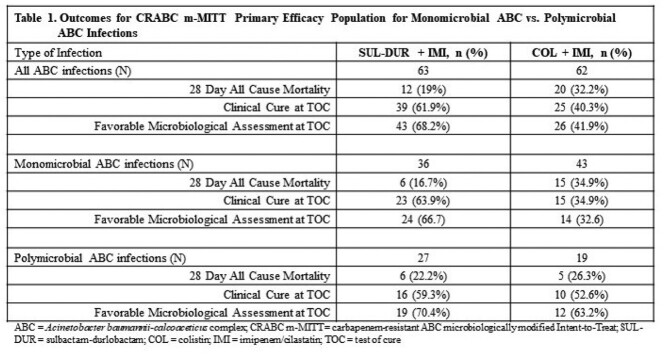

**Figure:**
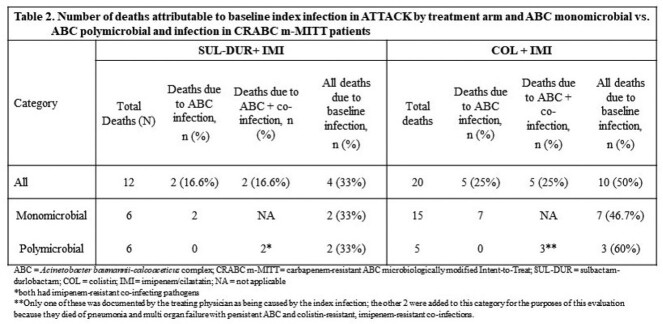

**Conclusion:**

SUL-DUR was equally efficacious in patients with monomicrobial vs. polymicrobial ABC baseline infections and an equal percentage of deaths (33%) were attributable to the index infection in each sub-group. Although fewer patients in the COL arm died with polymicrobial ABC infections (5 of 20, 25%) than monomicrobial ABC infections (15 of 20, 75%), 60% of the former (3 of 5) were attributable to polymicrobial ABC infections while 46.7% of the latter (7 of 15) were attributable to monomicrobial ABC infections. These results suggest that, if approved, the use of SUL-DUR plus a carbapenem could be an effective approach to treat polymicrobial infections that include ABC.

**Disclosures:**

**Alita Miller, PhD**, Entasis Therapeutics: employee|Entasis Therapeutics: Stocks/Bonds **Sarah McLeod, PhD**, Innoviva Specialty Therapeutics: Employee|Innoviva Specialty Therapeutics: Employee|Innoviva Specialty Therapeutics: Stocks/Bonds|Innoviva Specialty Therapeutics: Stocks/Bonds **Adam B. Shapiro, Ph.D**, Innoviva Specialty Therapeutics: Employee|Innoviva Specialty Therapeutics: Stocks/Bonds **Khurram Rana, PharmD**, Innoviva Specialty Therapeutics: Employee|Innoviva Specialty Therapeutics: Stocks/Bonds **David Altarac, MD, MPA**, Entasis Therapeutics: Full Time Employee

